# Preferences and Determinants of End-of-Life Care Among Older Chinese Americans

**DOI:** 10.1093/geroni/igab046.1378

**Published:** 2021-12-17

**Authors:** Dexia Kong, Elissa Kozlov, XinQi Dong

**Affiliations:** 1 The Chinese University of Hong Kong, Hong Kong , Hong Kong; 2 Rutgers University, Piscataway, New Jersey, United States; 3 Rutgers University, Rutgers Institute for Health, New Jersey, United States

## Abstract

End-of-life (EOL) care awareness and practice remain particularly low among older Chinese Americans. More empirical evidence regarding EOL is needed to develop culturally-relevant interventions to promote EOL engagement in this minority population. Using population-specific data, this study investigates preferences and associated sociodemographic and health determinants related to EOL among older Chinese Americans. Data were from the Population-based Study of Chinese Elderly in Chicago (collected 2017-2019, N=3,124). Linear and logistic regressions were conducted. Of the sample, 46.1% considered EOL care planning as important or somewhat import. Nearly 22% had EOL discussions with families. The most preferred EOL locations were home (43.7%), hospital (35.5%), nursing home (10.1%), and hospice (4.3%). Overall, 47.1% perceived EOL care as family decisions, 39.6% regarded EOL care as personal decisions, 7.5% preferred children to make EOL decisions, and 3.3% preferred a spouse to make EOL decisions. Chinese older adults who were female (B=0.10, p<0.01), married (B=0.11, p<0.01), had higher education (B=0.02, p<0.001), acculturation level (B=0.02, p<0.001), and religiosity (B=0.12, p<0.001), and more chronic conditions (B=0.05, p<0.001) were more likely to consider EOL as important. Those with older age [Odds Ratio (OR)=1.02, 95% Confidence Interval (CI)=1.01-1.03], female gender (OR=1.44, 95% CI=1.18-1.77), higher levels of education (OR=1.02, 95% CI=1.01-1.04), acculturation (OR=1.04, 95% CI=1.01-1.06), and religiosity (OR=1.11, 95% CI=1.02-1.21), longer U.S. residence (OR=1.02, 95% CI=1.01-1.03), and more chronic conditions (OR=1.13, 95% CI=1.06-1.21) were more likely to have discussed EOL preferences with their families. Study findings underscore low engagement in EOL planning in this population and the need for culturally-appropriate interventions.

